# Immune–microbe interactions in pediatric ulcerative colitis: clinical features, pathogenesis, and novel targeted therapeutic strategies

**DOI:** 10.3389/fimmu.2026.1948185

**Published:** 2026-09-14

**Authors:** Qiang Zhang, Lili Liang, Bo Ding, Kairan Zhou, Mengxue Hao, Kai Jiang, Xiaochun Feng, Jinhua Feng

**Affiliations:** 1 Changchun University of Chinese Medicine, Changchun, Jilin, China; 2 The Affiliated Hospital to Changchun University of Chinese Medicine, Changchun, Jilin, China

**Keywords:** B cells, intestinal epithelial barrier, macrophages, pediatric ulcerative colitis, T cells

## Abstract

Pediatric ulcerative colitis (PUC) is a chronic, immune-mediated inflammatory disease that exhibits different clinical characteristics and pathogenesis from those in adults. PUC is more severe and has a wider lesion range. The main manifestations include diarrhea, bloody stool, abdominal pain, and tenesmus, as well as systemic symptoms such as weight loss and anemia. However, the current treatment regimens for children with PUC cannot meet the increasing clinical cure demands, which is due to insufficient understanding of the pathogenesis of PUC. The pathogenesis of PUC is believed to be caused by the interaction of genetic and environmental factors, leading to an excessive and dysregulated immune response of the intestinal mucosa to microorganisms. This review summarizes the epithelial barrier defect, excessive innate immune activation, adaptive immune dysregulation, and associated cytokine dysregulation in the pathogenesis of PUC. Moreover, the potential therapeutic effects of new immunomodulatory therapies, including microbial therapy and anti-integrin biological agents, on PUC are summarized. The aim is to deepen the understanding of the pathogenesis and treatment mode that distinguish PUC from adults and promote precise treatment for PUC.

## 1 Introduction

Pediatric ulcerative colitis (PUC) represents a chronic and recurrent subtype of inflammatory bowel disease, whose incidence has been gradually increasing among children and adolescents. Compared with adult patients, pediatric individuals affected by UC typically exhibit a more severe clinical phenotype and a wider range of intestinal lesions (1). The median age at diagnosis falls between 12 and 15 years, and the overall gender distribution is relatively balanced, even though certain investigations have noted a mild male preponderance (2). Within the disease spectrum of pediatric ulcerative colitis (PUC), very early-onset UC (VEO-UC), defined as onset before 6 years of age, represents an etiologically distinct subgroup (3). Unlike older children, VEO-UC is frequently associated with monogenic mutations, among which defects in the IL-10 and IL-10 receptor (IL-10R) signaling pathway are the most representative (4). In children with VEO-IBD, *IL10RA* mutations are the most common, with the hotspot mutation c.301C>T (exon 3, p.R101W) and c.537G>A (exon 4, p.T179T-splicing mutation) (5). VEO-UC provides a valuable evidence for understanding the critical role of IL-10 in intestinal immune homeostasis (6). A considerable number of studies have confirmed that children with UC commonly present prominent clinical symptoms, such as abdominal pain, bloody diarrhea, and tenesmus (7). Nevertheless, a subset of pediatric patients may show growth retardation, systemic inflammatory reactions, or extra-intestinal complications as the primary clinical presentation. Of note, extra-intestinal manifestations represent a key clinical feature in PUC that deserves clinical attention. While these manifestations are less common than in Crohn’s disease, they may affect multiple organ systems including the joints, skin, and eyes (8). As an example, one study conducted in pediatric Crohn’s disease patients identified growth retardation and oral mucosal ulcers as the most frequent extra-intestinal involvements (9). Available evidence suggests that extensive colitis (pancolitis) is more prevalent in the pediatric population, and the disease activity at initial diagnosis is usually more intense. These characteristics are closely associated with a lower response rate to conventional treatments and an increased risk of hospital admission (10). Accordingly, a comprehensive insight into the unique clinical characteristics of PUC is essential for achieving early identification, accurate risk stratification, and rational improvement of treatment regimens.

Clinicians establish the diagnosis of PUC through a multidimensional evaluation system that combines clinical manifestations, endoscopic morphological changes, histopathological results and imaging examinations. Even so, whether endoscopic lesions can be well matched with pathological diagnostic outcomes in children is still controversial in current clinical research (7). Cross-age cohort analyses have verified that the consistency between typical endoscopic signs (including mucosal hyperemia and erythema, disappearance of normal vascular texture, heightened mucosal fragility, as well as mucosal erosion and ulcer formation) and pathologically verified UC lesions is not completely consistent. This inconsistency is especially prominent in early lesions, mild inflammatory changes and atypical pathological manifestations (7). In terms of evaluating disease activity, the Pediatric Ulcerative Colitis Activity Index (PUCAI) is the most commonly adopted scoring tool. Plenty of validation studies have proven its strong correlation with objective laboratory and endoscopic indicators, such as endoscopic inflammatory grading, fecal calprotectin and serum C-reactive protein, which makes it a credible reference basis for clinical treatment arrangement (11). Despite its practical value in clinical practice, PUCAI is merely a comprehensive scoring system built on clinical symptoms, unable to interpret the biological pathways responsible for heterogeneous disease progression. For this reason, in-depth exploration of the pathogenic mechanisms, especially the immune signaling pathways that differentiate childhood-onset UC from adult-type disease, is of great necessity. Such research will help improve diagnostic precision, forecast long-term disease progression and formulate more standardized and individualized treatment plans.

The pathogenesis of PUC involves a complex interplay of multiple factors, including genetic predisposition, intestinal microbial dysbiosis, impaired epithelial barrier function, and dysregulated host immune reactions (12). A widely accepted theoretical framework suggests that the core mechanism underlying chronic intestinal inflammation in UC is an abnormal mucosal immune response directed against commensal microbes, which is accompanied by coordinated dysfunction in both the innate and adaptive immune branches (13). With regard to innate immunity, intestinal epithelial cells in children diagnosed with UC show abnormally high expression of interleukin-15 (IL-15), a multifunctional pro-inflammatory cytokine. This cytokine promotes the activation of mucosal lymphocytes and suppresses their apoptotic process, ultimately triggering pathological changes in the epithelial structure (12). In the adaptive immune system, UC has long been regarded as a Th2-dominated inflammatory condition (14). However, recent studies have revealed that Th1 polarization mediated by interferon-γ (IFN-γ) also participates in the pathogenesis of PUC (15, 16). In PUC patients, the inflamed colonic mucosa exhibits elevated IFN-γ levels alongside IL-17A (15), and mucosal expression of the Th1 signaling molecule STAT-4 is significantly enhanced during the acute phase (16). These findings indicate that PUC is characterized by a dysregulated Th1 and Th17 cytokine response rather than a purely Th2-driven process (15).” In pediatric Crohn’s disease, it is accompanied by elevated expression of IFN-γ and granulocyte-macrophage colony-stimulating factor (GM-CSF) in the ileal mucosa, as well as reduced secretion of α-defensins (DEFA5 and DEFA6) produced by Paneth cells (17). While similar age-related analyses in colonic mucosa of PUC patients are more limited, available evidence suggests that colonic immune maturation may also shape disease presentation in children. For instance, transcriptomic profiling of mucosal biopsies from pediatric UC patients has revealed age-dependent clustering of inflammatory gene expression, with certain immune pathways becoming more pronounced in older children (18). These observations support the notion that age-associated maturation of the mucosal immune system-both in the small intestine and the colon-can influence IBD phenotype, highlighting the need for further pediatric-specific investigations in colonic tissue to better understand PUC pathogenesis.

Meanwhile, the gut microbiota, which acts as both an initiator and a target of host immune reactions, undergoes marked alterations in community structure among children with active ulcerative colitis. Specifically, increased mucosal colonization by *Lactobacillus* and *Enterococcus* has been detected, while anti-inflammatory commensal genera such as *Faecalibacterium prausnitzii* and *Blautia* are markedly reduced (19). This state of microbial dysbiosis is further supported by the observation that children with active UC exhibit elevated serum IgG responses against gut microbial antigens, which show a significant positive correlation with the severity of mucosal inflammation (20). In addition to these mechanistic findings, the measurement of immune-associated molecules has shown considerable clinical value in the diagnosis and prognostic assessment of PUC. Analysis of public transcriptomic datasets has demonstrated significantly higher mRNA and protein expression of key immune markers, including CD27, CD20 and myeloperoxidase (MPO), in inflamed intestinal tissues from pediatric patients (21). Moreover, serum and fecal biomarkers display strong associations with disease activity. C-reactive protein (CRP) and the Ulcerative Colitis Endoscopic Index of Severity (UCEIS) are closely correlated with patient-reported outcomes evaluated by the TUMMY-UC scale (r = 0.70). Meanwhile, fecal calprotectin, a commonly used non-invasive indicator, shows a correlation coefficient of 0.43 with endoscopic scores (1).

Taken together, the pathological progression of PUC is tightly associated with gut microbial imbalance, which arises from the interactive effects of genetic susceptibility and external environmental triggers. Its characteristic immune pathological manifestations cover excessive activation of intestinal epithelial cells and dendritic cells, a pro-inflammatory cytokine network coordinated by IL-15, TNF-α and IFN-γ, alongside insufficient production of defensins secreted by Paneth cells. Systematically unraveling such immune pathological pathways can lay a solid theoretical basis for designing precise treatment regimens centered on regulating mucosal immune function and intervening against intestinal flora disorders.

For this review, we performed a comprehensive literature search in PubMed using combinations of the following keywords: ‘pediatric ulcerative colitis,’ ‘very early onset inflammatory bowel disease,’ ‘intestinal epithelial barrier,’ ‘tight junctions,’ ‘mucosal immunity,’ ‘innate immunity,’ ‘macrophages,’ ‘dendritic cells,’ ‘neutrophils,’ ‘T cells,’ ‘B cells,’ ‘Th17,’ ‘Treg,’ ‘cytokines,’ ‘IL-10,’ ‘IL-17A,’ ‘IFN-γ,’ ‘IL-13,’ ‘gut microbiota,’ ‘dysbiosis,’ ‘fecal calprotectin,’ ‘prediagnostic phase,’ ‘multi-omics,’ and ‘biologic therapy.’ The search was restricted to English-language articles published up to August 2026. In addition, relevant clinical information was supplemented by consulting authoritative online resources, including Medscape. Additional relevant studies were identified by manual screening of reference lists of retrieved articles and key reviews. Priority was given to studies directly addressing the immunological mechanisms, clinical features, and therapeutic strategies of pediatric ulcerative colitis.

## 2 Clinical features of PUC

PUC represents one of the main subtypes of inflammatory bowel disease (IBD) affecting children. As a chronic and recurrent inflammatory condition affecting the colon, PUC in pediatric patients displays remarkable phenotypic heterogeneity and unique age-related clinical characteristics (22). The most representative clinical features of childhood UC are mainly concentrated in the gastrointestinal system (23). Mucosanguineous diarrhea, defined as stool containing mucus and visible blood, is widely recognized as the most typical clinical manifestation (22). Abdominal pain is another frequent symptom, usually described as cramping pain confined to the lower abdomen or diffusely distributed throughout the abdominal cavity (24). During periods of active inflammation, affected children may develop weight loss, which results from the combined effects of impaired nutrient absorption, decreased oral intake, and increased catabolic status (25). Notably, the diagnostic value of these typical symptoms in the pediatric population remains controversial. Children from different age groups often present with variable combinations of symptoms and differing severity levels, which further increases the difficulty of early diagnosis (22).

Clinical manifestations in PUC extend far beyond the gastrointestinal tract. Owing to the underlying disease process and persistent systemic inflammation, insufficient energy intake is extremely common. A prospective study conducted in Spanish children with UC reported that 55.31% of patients failed to achieve adequate energy intake, rendering weight loss and growth retardation frequent comorbid complications (26). Venous thromboembolism (VTE) constitutes a severe yet underrecognized extra-intestinal complication. Among pediatric IBD patients who develop VTE, up to 70% of cases are associated with UC, and more than half of thrombotic episodes occur within the first year after diagnosis (27). Renal dysfunction represents another important extra-intestinal issue that requires close monitoring. It may develop either as a direct consequence of UC pathogenesis or secondary to medications commonly used in treatment, such as 5-aminosalicylates and tacrolimus (28). In addition, children with comorbid UC and celiac disease show a significantly elevated risk of delayed puberty (14.9% versus 3.2%) (29), implying that overlapping autoimmune disorders may amplify the adverse impacts on pediatric growth and maturation.

In conclusion, the clinical spectrum of PUC involves multiple dimensions, including gastrointestinal manifestations, nutritional disorders, extra-intestinal organ involvement, and psychosocial status. Early identification of these variable clinical features plays a critical role in achieving timely diagnosis, formulating personalized treatment strategies, and ultimately improving long-term prognosis and quality of life.

From an epidemiological perspective, PUC exhibits distinct features relative to adult-onset disease, including more extensive intestinal lesions, more severe clinical phenotypes, greater demand for intensive treatment, and a higher incidence of postoperative complications (30). Given the globally rising incidence of UC (31), a deeper understanding of the intestinal immune features and pathogenic mechanisms underlying PUC is of significant value for identifying biomarkers that can predict disease severity and therapeutic response.

## 3 Immunological features of the pediatric intestine and their role in PUC pathogenesis

The intestinal immune system in children differs markedly from that in adults, exhibiting a highly dynamic and stage-specific developmental trajectory. During early life, the gut serves not only as the primary site for nutrient digestion and absorption but also as a critical ecological niche where the immune system establishes key interactions with the external environment-particularly the gut microbiota (32, 33). The intestinal immune system of neonates and infants displays characteristics distinctly different from those of adults, with core features of remarkable “plasticity” and “activation” (34). Functional follicular helper T cells, identified as CXCR5^+^PD-1^++^ CD4^+^ T cells, are not present in the fetal intestine. These cells undergo rapid expansion after birth and peak during infancy, with levels significantly exceeding those detected in adult individuals (35). Meanwhile, the maturation of the intestinal immune system relies heavily on dynamic interactions with the gut microbiota. The relatively elevated intestinal permeability observed during the neonatal period supports the translocation of microbial components and antigens to submucosal immune populations, thereby facilitating immune education and developmental maturation (36). Of note, the developmental status of the intestinal immune system differs substantially across age groups and directly shapes the clinical phenotype of inflammatory bowel disease-older children aged 10 to 16 years with Crohn’s disease more commonly exhibit ileal involvement with a prominent Th1-polarized immune response, whereas younger children under 10 years of age show a higher proportion of colonic involvement, a disparity closely linked to the maturation status of ileal Peyer’s patches and Paneth cell function (10) (17).

Compared with adult UC, PUC typically presents with more extensive disease distribution (predominantly pancolitis), greater disease activity, and a higher requirement for treatment escalation, suggesting that the immunopathological mechanisms of PUC possess unique age-related characteristics (10). PUC development is driven by intricate disruptions in the intestinal immune network, primarily manifested as a self-amplifying vicious cycle stemming from compromised intestinal epithelial barrier integrity and dysregulated mucosal immune activation (37). Emerging research has validated that synergistic dysfunction of innate and adaptive immunity triggers disease initiation and facilitates its continuous progression (**Figure 1**), involving abnormal functional status of diverse immune cell populations—including intestinal epithelial cells, macrophages, dendritic cells, T lymphocytes, and B lymphocytes—coupled with excessive activation of downstream cytokine and chemokine signaling cascades (such as IL-1β, TNF-α, IL-6, IL-17A, and IFN-γ) (15, 38, 39).

**Figure 1:**
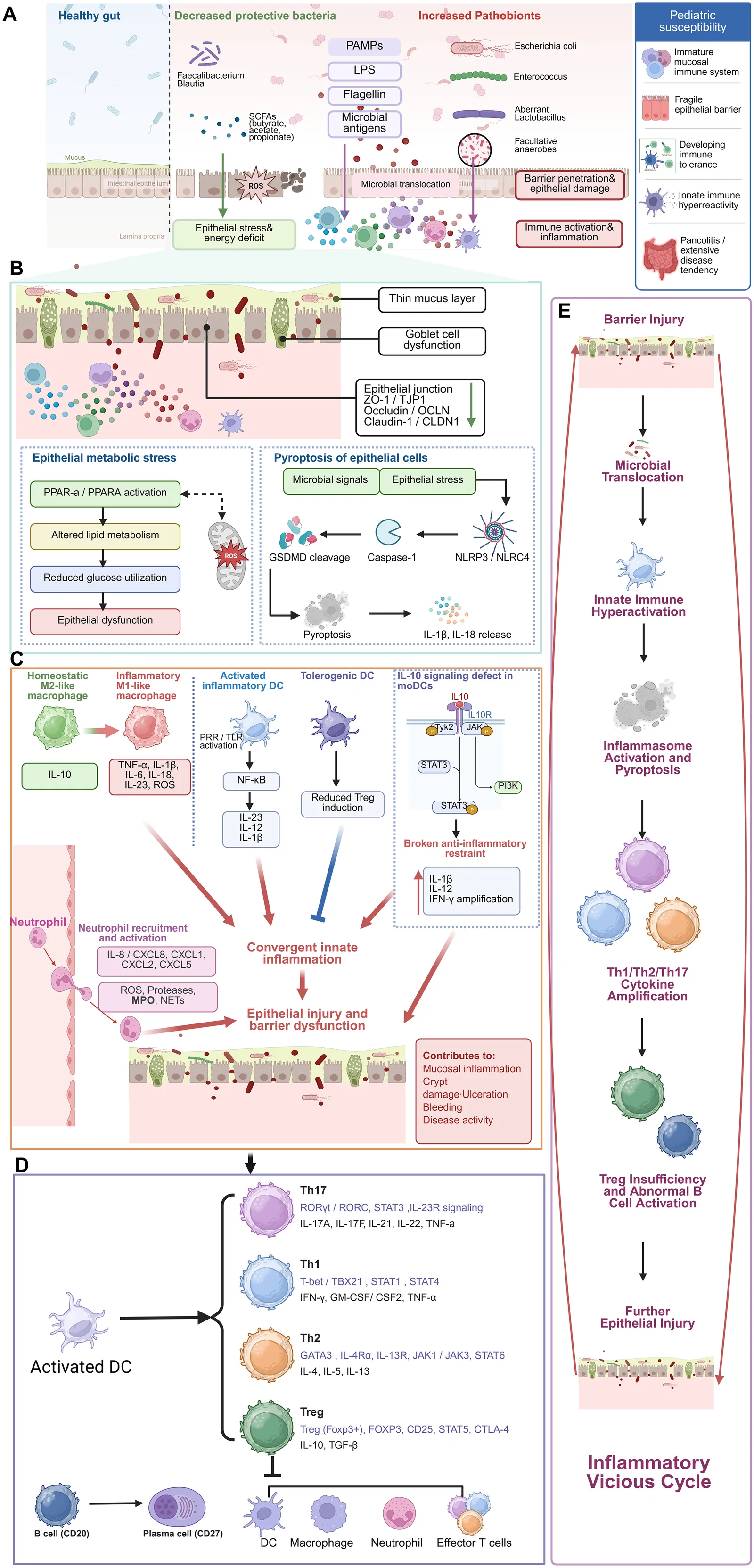
Pathogenesis of pediatric ulcerative colitis: epithelial barrier defects and immune dysregulation drive mucosal inflammation. **(A)** Within the colonic lumen, gut microbial dysbiosis is marked by the overgrowth of opportunistic pathogenic bacteria (e.g., *Enterococcus*, *Escherichia coli*) and the depletion of beneficial commensal species such as *Faecalibacterium* and *Blautia*. **(B)** Decreased expression of tight junction proteins (ZO-1, Occludin, Claudin-1) and defective antimicrobial defense function disrupt the structural integrity of the epithelial barrier, giving rise to elevated intestinal permeability and enhanced microbial translocation. **(C)** Translocated microbial components engage pattern recognition receptors (PRRs) on antigen-presenting cells (macrophages and dendritic cells) residing in the lamina propria. This signaling activation drives macrophage polarization toward the pro-inflammatory M1 phenotype, which secretes IL-1β, IL-18 and other inflammatory cytokines, and further mediates neutrophil recruitment. **(D)** Activated dendritic cells promote the differentiation of naïve CD4^+^ T cells into pro-inflammatory Th1, Th17 and Th2 subsets. This process is accompanied by functional insufficiency or impaired immunosuppressive capacity of regulatory T cells (Treg), as well as aberrant activation of humoral immunity (B cells/plasma cells), all of which jointly contribute to intestinal epithelial injury. **(E)** These interconnected pathways form a positive feedback loop that sustains chronic inflammation.

The following sections discuss in detail how the developmental immaturity and fragility of the pediatric intestinal epithelial barrier, the hyperreactive state of innate immunity, and the dysregulated adaptive immune responses—along with their associated cytokine networks—collectively lay the immunological foundation for the distinctive pathogenesis of PUC.

### 3.1 Intestinal epithelial barrier: developmental immaturity and injury in PUC

The intestinal mucosal barrier in children is not yet fully developed, and its defensive capacity is inferior to that of adults, constituting a major reason for the increased susceptibility of children to intestinal infections (40, 41). The intestinal barrier comprises physical, chemical, immunological, and microbial components, the functional maturation of which depends on progressive postnatal development (42). At the level of the physical barrier, the mucus layer covering the intestinal epithelium is markedly reduced in preterm infants, allowing potentially harmful molecules that would otherwise be segregated to come into direct contact with the underlying epithelium, thereby triggering tissue injury (43–45). The intact expression of tight junction proteins-including ZO-1, occludin, and claudin-1-between intestinal epithelial cells is essential for maintaining barrier integrity (46, 47). During development, the assembly and functional regulation of these proteins may not yet have reached homeostatic equilibrium, rendering the pediatric gut relatively hyperpermeable (48). Studies have shown that intestinal epithelial cells rely on tight junctions (TJ) and adherens junctions to maintain seal integrity, and the maturity of these junctions is lower during childhood (49, 50). Microbial colonization also plays a key role in this process-in preterm infants, the rapid decline in intestinal permeability during the first two weeks of life correlates positively with a significant increase in microbial community diversity, suggesting that barrier maturation is closely intertwined with microbial establishment (51).

This inherently fragile barrier becomes further compromised in the context of PUC. Pediatric patients with UC, especially young children, commonly suffer from more widespread and severe colonic inflammatory lesions. This unique clinical phenotype is tightly associated with the immature developmental status and inherent fragility of the intestinal epithelial barrier in children (10). Relevant investigations based on colon epithelial organoids (colonoids) isolated from PUC patients in active disease stages have confirmed that intestinal epithelial cells undergo excessive metabolic activation and cellular stress during differentiation, alongside abnormal upregulation of the peroxisome proliferator-activated receptor alpha (PPAR-α) signaling cascade. In the context of PUC, activated PPAR-α signaling inhibits cellular glucose metabolism and aggravates metabolic stress responses. This pathological process ultimately induces epithelial cell damage and promotes the secretion of pro-inflammatory chemokines (52). Thus, epithelial metabolic disorders are not merely a secondary inflammatory consequence but serve as a pivotal pathogenic factor that destabilizes barrier integrity.

Immune barrier dysfunction constitutes another hallmark of PUC that profoundly disrupts intestinal microenvironmental homeostasis (21, 53). Serum IgG reactivity to gut microbes is significantly elevated in children with active UC and correlates positively with mucosal inflammation severity (20). Accumulation of IgG antibody complexes targeting specific microbial taxa, such as those of the *Lactobacillaceae* family, may suppress complement activation and perturb normal immune responses to the microbiota, thereby contributing to mucosal immune barrier dysfunction. Concurrently, densities of CD27- and CD20-positive immune cells (including B cells and plasma cells) as well as MPO-positive cells are markedly increased in diseased intestinal tissues, indicating overactivation of local humoral immunity and neutrophil-mediated responses that further aggravate epithelial injury (21).

Microbial barrier dysregulation adds another layer of complexity in PUC. Unlike in adult patients, the colonic mucosa-associated microbiota in children with UC-particularly those with pancolitis-exhibits a disordered homogenized distribution, with compositional features converging toward those of luminal microbiota in feces (19). As disease activity intensifies, opportunistic taxa such as *Lactobacillus* and *Enterococcus* increase in abundance, while protective genera including *Faecalibacterium* and *Blautia* decline markedly (19). This upward shift of microbial distribution toward proximal segments and excessive colonization of pathogenic strains disrupt the protective effects of symbiotic flora. Meanwhile, altered microbial metabolite profiles—particularly decreased synthesis of short-chain fatty acids—exacerbate energy deficiency and metabolic disorders within intestinal epithelial cells (7, 53–55).

Taken together, epithelial barrier damage in PUC constitutes a self-perpetuating pathological loop driven by intertwined metabolic, immune, and microbial abnormalities. Metabolic anomalies directly undermine physical barrier integrity and trigger massive secretion of pro-inflammatory mediators, while subsequent dysregulated humoral immune activation and bacterial translocation further amplify mucosal injury (52, 56).

### 3.2 Innate immunity: hyperreactivity and dysregulation

In early childhood, the intestinal innate immune system exhibits distinctive characteristics, most notably a state of heightened immune reactivity. This developmental trait shapes children’s ability to resist pathogenic microbes while simultaneously rendering them more vulnerable to intestinal inflammatory disorders. Preterm infants and neonates frequently present with a “leaky gut”—increased intestinal epithelial permeability—that allows luminal microorganisms, antigens, and metabolites to readily traverse the barrier and activate submucosal innate immune cells (57). A variety of luminal factors, including commensal metabolites, bile acids, and neuropeptides, regulate the migration and function of innate immune cells through neuro-immune cell units; dysregulation of these circuits may precipitate inflammatory bowel disease, irritable bowel syndrome, and intestinal allergies (58). Notably, studies in murine models of neonatal necrotizing enterocolitis (NEC) have demonstrated that the NLRP3 inflammasome is activated in the neonatal intestine, leading to caspase-1-mediated Gasdermin D cleavage, pyroptosis, and the release of large amounts of pro-inflammatory cytokines such as IL-1β and IL-18 (59). Similarly, in a mouse model carrying a gain-of-function mutation in NLRC4 (NLRC4 V341A) that closely recapitulates human autoinflammation with infantile enterocolitis (AIFEC), hyperactivation of the NLRC4 inflammasome in the neonatal gut epithelium triggers caspase-1 activation, GSDMD cleavage, and pyroptosis, accompanied by elevated IL-1β and IL-18 production (60). The hyperresponsiveness of this pathway is especially evident in children with early-onset enterocolitis or viral myocarditis, suggesting that innate immune sensors in the pediatric gut have not yet established sufficient negative feedback inhibitory mechanisms (61, 62). Collectively, this overactive innate immune status stems from three interrelated factors: incomplete maturation of intestinal physical barrier structures, overactivated inflammasome signaling cascades, and the cross-talk between gut flora imbalance and aberrant immune cell function.

This hyperreactive baseline sets the stage for the more profound innate immune dysregulation observed in PUC. Aberrant macrophage activation serves as a core trigger for persistent intestinal inflammatory injury (63). Under physiological conditions, intestinal macrophages adopt a tolerogenic, M2-skewed phenotype, sustaining epithelial barrier stability and immune homeostasis through IL-10 secretion (63, 64). In contrast, studies in a juvenile murine colitis model have demonstrated that probiotic intervention with Pediococcus pentosaceus CECT8330 can simultaneously remodel the gut microbiota and reprogram macrophages from a pro-inflammatory M1 phenotype toward an anti-inflammatory M2 phenotype, suggesting a link between microbial modulation and macrophage polarization in the inflamed gut (65). Although these findings derive from a DSS-induced colitis model rather than PUC patients directly, they suggest a plausible mechanism whereby microbial signals influence macrophage function in the inflamed gut. Inflamed colonic tissues harbor specific commensal *Escherichia coli* strains that employ virulence factors such as the type III secretion system to trigger assembly and activation of NLRC4, caspase-8, and caspase-1/11 inflammasome complexes, promoting IL-18 maturation and release from intestinal epithelial cells (63, 66). IL-18 subsequently facilitates the proliferation of Th1 cells and NKp46^+^ IFN-γ-expressing cells, both of which drive macrophage polarization toward the pro-inflammatory M1 phenotype (63). Once polarized, M1 macrophages robustly secrete TNF-α, IL-1β, and IL-6, forming a positive feedback loop that continuously amplifies local intestinal inflammation and ultimately results in irreversible tissue damage (67, 68).

Dendritic cells (DCs) play a central role in initiating and regulating intestinal immune responses (69). Under steady-state conditions, intestinal DCs tend to induce regulatory T cell differentiation and maintain immune tolerance; upon exposure to pro-inflammatory signals in an inflammatory milieu, however, DCs shift toward promoting effector T-cell responses (70). In PUC, aberrant signals from dysbiotic microbiota-such as pathogen-associated molecular patterns-activate pattern recognition receptors on DCs, driving them to secrete inflammatory cytokines including IL-23 (71, 72). For instance, a study in a cohort of children with Hirschsprung disease (HSCR) demonstrated a functional crosstalk between cholinergic nerve fibers and mucosal macrophages, in which the presence of mucosal innervation correlated with reduced macrophage-dependent IL-23 expression, thereby dampening Th17-type immune responses (73). Thus, in PUC, when DCs encounter aberrant pro-inflammatory signals from the dysbiotic microbiota, their capacity to secrete IL-23 is enhanced, promoting the differentiation and expansion of pathogenic Th17 cells (74, 75).

Neutrophils represent another key effector arm of innate immunity in PUC. Their excessive infiltration is a hallmark of mucosal injury in active disease (76). Neutrophil recruitment and activation are under stringent physiological regulation in healthy tissues (77), but this regulatory balance is drastically overactivated in UC. Reduced cholinergic innervation in the intestinal mucosa-a feature observed in hypoganglionic Hirschsprung disease-can markedly upregulate IL-8 secretion by intestinal epithelial cells. This abnormal IL-8 elevation correlates closely with increased risk of enterocolitis and aggravated inflammatory cellular infiltration (78). As a key chemokine, IL-8 directs the directional migration and functional activation of neutrophils (79). Children with Hirschsprung disease (HSCR) are prone to severe Hirschsprung-associated small intestinal colitis (HAEC) (78). This is because cholinergic signals inhibit the production of IL-8 by intestinal epithelial cells (IEC) through the α7 nicotinic acetylcholine receptor (α7nAChR). In children with sparse mucosal cholinergic nerve fibers (fiber-low), the level of pro-inflammatory factor IL-8 produced by intestinal epithelial cells (IEC) significantly increases, and this increase in IL-8 is associated with a higher incidence of HAEC (78). This suggests that even subtle changes in intestinal neural innervation in PUC may contribute to the formation of an inflammatory microenvironment and the persistent infiltration of neutrophils. Future studies need to further explore whether such mechanisms play a role in PUC.

Taken together, the innate immune system in PUC is characterized by a developmentally programmed hyperreactivity that is intrinsic to the pediatric gut, upon which an additional layer of dysregulation-including aberrant macrophage polarization, DC-mediated Th17 skewing, and excessive neutrophil recruitment-is superimposed in the inflammatory milieu. This two-level abnormality establishes the innate immune compartment as a central driver of mucosal inflammation in PUC.

### 3.3 Adaptive immunity: the double-edged sword and aberrant activation

The intestinal adaptive immune response in early childhood displays a distinctive dual regulatory property, requiring a precise dynamic equilibrium between fending off invasive pathogens and establishing immune tolerance toward innocuous antigens. Unlike adult counterparts, gut-associated lymphoid tissue (GALT) in pediatric individuals elicits markedly stronger immune reactions when exposed to unfamiliar antigens (80). This hyperreactivity facilitates the establishment of extensive protective immunological memory, yet simultaneously increases the risk of autoimmunity and excessive inflammatory reactions. Although large numbers of effector-memory phenotypic CD8^+^ T cells are already present in the infant gut, their cytotoxic functions-including cytokine and granzyme B production-are significantly weaker than those in adults, and these effector cells highly express the inhibitory molecule PD-1, resulting in limited antiviral capacity (81). This functionally attenuated CD8^+^ T-cell response helps maintain immune tolerance to the vast array of new antigens encountered postnatally, but also constitutes an intrinsic immunological basis for the heightened susceptibility of infants to gastrointestinal viral infections (81). Similarly, in Crohn’s disease, younger children (<10 years) exhibit a stronger Th1-associated IFN-γ signature and more pronounced Paneth cell α-defensin downregulation compared with older children, suggesting that the younger the host, the more vigorous the effector arm of adaptive immunity may be, yet the less mature its negative regulatory mechanisms (17).

This finely balanced system, however, is profoundly disrupted in children with UC. Studies in pediatric UC patients have demonstrated that CD4^+^ effector T cells-particularly Th1 and Th17 subsets-play a central role in driving intestinal inflammation (82, 83). Under normal conditions, the host employs multiple tolerogenic mechanisms, including T-cell apoptosis, to maintain immune homeostasis (84). Studies in human intestinal T cells have shown that apoptosis is required to induce tolerance and maintain a state of ‘physiological’ inflammation in the intestine, where mucosal T cells are constantly exposed to high antigenic pressure from luminal antigens (84). Mechanistic studies in murine models and human T cell lines have revealed that retinoic acid, through binding to retinoic acid receptor alpha, induces high expression of the purinergic receptor P2X7 on CD4^+^ effector T cells; activation of P2X7 effectively induces apoptosis in these cells, achieving “effector cell contraction” and thereby suppressing colitis development (85).

Accumulating evidence indicates that the intestinal mucosa of children with UC harbors a prominent and distinctive Th2-cell-driven immune activation profile, which differs from the immunopathology observed in adult patients and in other types of pediatric IBD (82). In diseased intestinal tissues, Th2 cells and their signature cytokines—including IL-4, IL-5, and IL-13—are markedly increased in abundance (82). Overexpression of these cytokines can directly act on intestinal epithelial cells, leading to disruption of epithelial barrier function, promotion of aberrant mucus secretion, and recruitment of eosinophils, thereby perpetuating chronic inflammation (86). Unconventional T-cell subsets such as γδ T cells and invariant natural killer T (NKT) cells also occupy a distinctive position in the immune network of PUC (87). Studies in adult UC patients have shown that the frequency of specific γδ T-cell subpopulations is significantly reduced in both peripheral blood and intestinal mucosa, whereas their apoptosis rate is abnormally increased, suggesting that these cell populations-which possess potent cytotoxic and immunoregulatory functions-are extensively depleted under disease conditions (88, 89).

The maintenance of intestinal immune microenvironment homeostasis is highly dependent on the generation and functional performance of regulatory T (Treg) cells (90). Under steady-state conditions, CD103^+^ dendritic cells in the gut transport microbiota-derived antigens to mesenteric lymph nodes and induce the differentiation of RORγt^+^Helios^-^ induced Treg (iTreg) cells-a process essential for suppressing aberrant immune responses against commensal bacteria (91). In PUC, however, this tolerogenic circuit is frequently disrupted. The CD40 signaling pathway, acting as a regulatory hub independent of microbial signals, promotes the emigration of CD103^+^ DCs from the lamina propria, thereby impairing iTreg cell induction. In mouse models, sustained CD11c-specific CD40 signaling results in a marked reduction of RORγt^+^Helios^-^ iTreg cells, accompanied by exacerbated Th1/Th17 inflammatory responses and even fatal colitis (91). These findings suggest that aberrant activation of the CD40L/CD40 signaling axis may represent a critical mechanism underlying the early-life breakdown of intestinal tolerance in PUC. In pediatric IBD patients, circulating Treg frequencies are increased at diagnosis-likely as a compensatory response to tissue inflammation—yet this elevated Treg density remains insufficient to effectively counteract intestinal inflammation (92). In PUC patients specifically, a study of pediatric UC patients demonstrated that Treg cell numbers are significantly decreased compared with healthy controls, and the Treg/Th17 ratio is markedly reduced (93). In this cohort, the percentage of peripheral CD4^+^FOXP3^+^ Tregs was significantly lower in UC patients than in healthy controls, and the Treg/Th17 ratio was decreased in IBD patients, with a strong correlation between the Treg/Th17 ratio and disease activity (93). These findings from pediatric UC patient cohorts establish that Treg deficiency and Treg/Th17 imbalance are characteristic features of PUC immunopathology. Concurrently, aberrant activation of Th17 cells further exacerbates this immune imbalance, with significant overexpression of IL-17A observed in the intestinal mucosa of children with UC (94).

B cells and their associated antibodies also play an indispensable role in the initiation, perpetuation, and exacerbation of PUC, particularly in children with higher disease activity (95). B-cell dysfunction is directly reflected in an aberrant humoral immune response to the gut microbiota (20). The proportion of IgG-coated gut microbiota is significantly elevated in children with active UC and correlates positively with the severity of mucosal inflammation (20). Transcriptomic and immunohistochemical analyses have further confirmed that mRNA and protein expression levels of the B-cell marker CD20 and the plasma cell marker CD27 are significantly increased in inflamed intestinal tissues (21). The abundant presence of CD27^+^ memory B cells and plasma cells in the intestinal mucosa of pediatric UC patients indicates persistent, locally antigen-driven B-cell differentiation and ongoing antibody production (38). Beyond antibody secretion, these infiltrating B cells also contribute to the inflammatory milieu by producing cytokines such as IL-6 and TNF-α (96), and by providing co-stimulatory signals, thereby directly modulating T-cell-mediated inflammatory responses (97). This bidirectional crosstalk establishes a B-cell–T-cell amplification loop that perpetuates mucosal inflammation (97, 98). Moreover, the distribution and cytokine profile of peripheral B-cell subsets are significantly perturbed in children with IBD (96). A study in 42 pediatric IBD patients found that the frequency of IL-10-producing B cells (B10 cells) is elevated and closely correlated with the Treg/Th17 ratio (99). In this pediatric cohort, the number of B10 cells was elevated in IBD patients, the ratio of Treg/Th17 decreased, and the Treg/Th17 ratio strongly correlated with B10 cells (99).

In summary, the adaptive immune system in PUC is marked by a triad of interconnected abnormalities: exaggerated effector T-cell responses (Th1, Th17, and Th2), impaired regulatory T-cell function, and aberrant B-cell-mediated humoral activation. Together, these disturbances dismantle the delicate balance between immune defense and tolerance that characterizes the normal pediatric gut, driving persistent mucosal inflammation and tissue injury in PUC.

### 3.4 Cytokine networks orchestrating mucosal inflammation

Classic research has long centered on individual pro-inflammatory or anti-inflammatory signaling cascades to interpret ulcerative colitis pathogenesis. However, accumulating contemporary evidence from pediatric UC patient cohorts demonstrates that PUC is governed by sophisticated crosstalk among diverse T lymphocyte subpopulations and their characteristic effector cytokines (82, 83, 100). In treatment-naïve pediatric UC patients, mucosal expression of type 2 immune response genes (IL5, IL13, IL13RA2) and type 17 immune response genes (IL17A, IL23) is significantly increased compared with patients with colon-only Crohn’s disease (82). Similarly, colonic biopsies from pediatric UC patients show elevated IL-17A expression in inflamed colon, with IL-17A levels positively correlated with IFN-γ, indicating a mixed Th1/Th17 inflammatory profile (83). IL-17A serves as a pivotal pro-inflammatory cytokine that drives intestinal inflammatory lesions in PUC (82). Transcriptomic analyses of colonic mucosal samples from treatment-naïve pediatric UC patients show marked upregulation of IL-17A, which aligns with findings observed in adult UC cases (15). This evidence indicates that the IL-17A-related signaling cascade is activated at the early stage of disease progression (94). Apart from its direct pro-inflammatory functions, IL-17A is capable of regulating the activity of intestinal visceral nociceptors through modulating the PI3K signaling axis, which accounts for gastrointestinal pain symptoms in UC patients (101). Previous studies have confirmed that γδT17 cells are a major cellular source of mucosal IL-17A (102, 103). The simultaneous increase of IL-17A expression and γδT17 cell abundance in inflamed colonic tissues further validates the indispensable role of Th17 immune responses in UC local immune injury (104).

In addition to Th17-mediated inflammation, Th1 immune responses driven by IFN-γ also actively participate in the pathological progression of pediatric ulcerative colitis. IL-10 acts as a core regulatory cytokine that tightly restrains excessive Th1 immune activation. Functional investigations based on cells isolated from patients carrying *IL10RA* deficiency or *STAT3* mutations have clarified that IL-10 exerts its inhibitory effect on IFN-γ production in an indirect manner. Instead of targeting T cells directly, IL-10 primarily modulates the biological function of monocyte-derived dendritic cells (moDCs) to suppress IFN-γ secretion (105). Defects in IL-10 signaling trigger abnormal overproduction of IL-1β in moDCs. The accumulated IL-1β further drives IFN-γ release from CD4^+^ T cells and promotes moDCs to generate more IL-12, forming a persistent pro-inflammatory positive feedback cycle that amplifies intestinal inflammation (105, 106). This unique immune dysregulation has been verified in specific pediatric IBD patient subgroups, which are characterized by upregulated IL-1β expression and impaired cellular sensitivity to IL-10-mediated immune suppression (105).

Th2-type cytokine signaling, with IL-13 as the prototypical effector molecule, also contributes to the initiation and progression of intestinal inflammatory injury in pediatric UC (82). In pediatric IBD, accumulating evidence indicates that IL-13 enhances the sensitivity of colonic afferent nerves through activation of the p38 MAPK signaling cascade, thus participating in the pathogenesis of visceral hypersensitivity (107). A study in a murine model demonstrated that IL-13-mediated sensitization of colonic afferents to noxious mechanical distension was dependent on p38 MAPK and JAK signaling, and that inhibition of p38 MAPK abolished this sensitization (107). These findings from animal models suggest that IL-13 and its downstream p38 MAPK pathway represent potential therapeutic targets for visceral pain in IBD. Within the intestinal immune microenvironment, the cooperative interaction between TGF-β and IL-6 drives the differentiation of non-pathogenic Th17 cell subsets (108). On the contrary, TGF-β acts synergistically with IL-1β to upregulate CCL20 expression, which may indirectly facilitate the directional chemotactic migration of Th17 cells toward cutaneous or intestinal mucosal tissues (109, 110). Disruption of this finely tuned equilibrium, particularly a functional shift in TGF-β signaling, can aggravate local inflammatory responses. Taken together, PUC is not driven by a single cytokine pathway. Instead, its pathogenesis relies on a highly intricate immune network shaped by dynamic crosstalk between innate and adaptive immune cells, pro-inflammatory mediators (IL-17A, IFN-γ, IL-13), and immunoregulatory molecules (IL-10, TGF-β).

In summary, the interplay between the immature intestinal mucosal barrier, hyperreactive innate immunity, and dysregulated adaptive immunity collectively forms the immunopathological foundation for the predisposition of PUC to early onset, extensive disease distribution, and a relapsing course.

## 4 Discussion and conclusion

PUC arises from the combined interplay of multiple pathogenic factors-genetic susceptibility, gut microbiota dysbiosis, and dysregulated mucosal immune activation-that act synergistically during critical developmental windows in early life (37, 111). While PUC shares core mechanistic underpinnings with adult-onset UC, notable divergences persist across disease severity, genetic predisposition profiles, gut microbial ecology, and immunometabolic features. A comprehensive understanding of these similarities and discrepancies is indispensable for formulating precise diagnostic criteria and tailored therapeutic strategies for pediatric patients.

In terms of clinical presentation and disease trajectory, PUC generally follows a more severe and extensive course compared with its adult counterpart, characterized by a higher rate of pancolitis, more frequent acute disease flares, and a larger proportion of patients requiring inpatient care (2, 10). This phenotypic disparity cannot be ascribed solely to delayed diagnosis or ascertainment bias. Instead, it most likely reflects an overamplified response of the still-developing pediatric intestinal immune system to external injurious stimuli during its highly plastic developmental phase (20). With respect to genetic background, although UC susceptibility loci related to immune regulation and intestinal barrier function are partially shared across age groups, PUC patients frequently carry distinct genetic variants that differ from those observed in adult populations. For instance, *NOD2* and *ATG16L1* mutations, which are prevalent in Western adult UC cohorts, are rarely detected in pediatric patients (112, 113). A GWAS of 466 PUC cases revealed that the HLA region on chromosome 6 explains almost the entire genetic association signal in PUC, with HLA-DRB10103 showing a particularly strong association (OR = 6.94, p = 1.92 × 10^-^¹³)-approximately twice the effect size observed in adult-onset UC (OR = 3.59) (114). Notably, while some HLA-DRB1 causal alleles are shared with adult UC, at least two signals appear to be unique to pediatric-onset disease (114). Furthermore, genetic architecture studies have demonstrated differences in the polygenic architecture of pediatric- versus adult-onset IBD, with a significant accumulation of rare and deleterious variants in affected children suggesting additional unexplained genetic contributions (115). By contrast, the ATG16L2 variant (rs11235604) identified in Korean children with Crohn’s disease points to the involvement of unique bacterial processing pathways in pediatric-onset inflammatory bowel disease (116, 117). In a cohort of 22 Korean children with infantile, very-early, and early-onset CD, the ATG16L2 variant was the most frequently occurring variant, carried by 41% of CD probands (116). Additionally, a study in the Chinese Han population confirmed ATG16L2 as a susceptibility gene for CD, with the rs11235604 SNP significantly associated with downregulation of ATG16L2 expression (117). These observations suggest that during early development, the interplay between genetic background and environmental triggers may differ substantially from that in adults, driving earlier-onset intestinal immune dysregulation.

The etiology of VEO-UC is closer to monogenic immune deficiency than to classical polygenic inflammatory bowel disease (118). Loss-of-function mutations in IL-10/IL-10R can lead to severe, refractory colitis presenting in infancy, with *IL10RA* mutations (c.301C>T, p.R101W; c.537G>A, p.T179T) predominating in Chinese patients, often accompanied by perianal lesions (119, 120). Such patients show limited response to conventional immunosuppressants and biologics, and allogeneic hematopoietic stem cell transplantation (HSCT) is currently the only potentially curative option (118, 120). Therefore, in children with onset before 6 years of age, severe disease, and poor response to conventional therapy, timely genetic testing should be performed to distinguish VEO-UC from typical PUC, providing a basis for early HSCT decision-making (6, 118).

In addition to genetic predisposition, early-life environmental exposures have emerged as critical determinants of IBD risk, operating through their impact on gut microbiota assembly and immune maturation during sensitive developmental windows (121). Perinatal and early childhood antibiotic exposure serves as a prime example: a nationwide Norwegian register study reported that antibiotic exposure before 2 years of age was associated with a 1.4-fold increased risk of pediatric IBD, and a systematic review confirmed a positive association between prenatal antibiotic exposure and IBD risk (122, 123). The effect of antibiotics is likely mediated through gut microbiome perturbation, which can impair immune tolerance establishment and promote persistent dysbiosis (121). Other early-life factors-including breastfeeding (protective), mode of delivery, and hygiene-related exposures-have also been linked to subsequent IBD risk (122). Collectively, these observations indicate that PUC pathogenesis is influenced by environmental factors operating long before clinical onset, particularly during the critical window of early-life microbiome and immune system development.

The aforementioned variants of genetic susceptibility genes provide a dysregulated initial set point for the intestinal immune system, yet genetic variation alone is insufficient to drive disease onset. Rather, it is the aberrant interpretation of and excessive response to microbial signals by the immune system during specific developmental windows in early life that constitute the direct factors of tissue injury. At the level of innate immunity, the developmental vulnerability of the pediatric intestinal epithelium and the hyperreactivity of innate immune cells act in concert, resulting in an excessively vigorous response to danger signals from commensal microbiota (124, 125). Unlike in adults, PUC exhibits a distinctive Th2-skewed immune profile, in which predominant activation of the IL-13/STAT6 pathway directly drives epithelial barrier disruption-a finding that partially explains the more extensive colonic involvement observed in children (82, 86). Concurrently, the functional immaturity of regulatory T cells and the relative deficiency of the IL-10 signaling pathway deprive the inflamed gut of effective negative regulation once inflammation is amplified, thereby contributing to the chronicity and refractoriness of the disease (92, 118, 126). These immunological characteristics collectively indicate that the pathogenesis of PUC is not a direct replication of the adult disease phenotype. Instead, it constitutes a distinct pathological process driven by the combined effects of early-life immune system developmental trajectories, genetic susceptibility, and microbial ecological disturbances. Comprehensive characterization of this age-dependent immune regulatory network can lay a foundational theoretical framework for developing therapeutic interventions targeting the crosstalk between innate and adaptive immune compartments.

PUC patients typically present with abrupt disease onset, rapid clinical progression, and widespread mucosal lesions, alongside a higher incidence of steroid resistance and elevated colectomy rates (127). Such clinical characteristics indicate that standard immunosuppressive treatment regimens-including 5-aminosalicylates, corticosteroids, and biologic agents-often exhibit limited therapeutic efficacy or carry an elevated risk of infectious complications in pediatric populations, attributable to the distinct immunological heterogeneity of their developing immune system (37, 128). At a deeper mechanistic level, these observations suggest that immune dysregulation in PUC does not merely reflect quantitatively enhanced inflammatory responses. Instead, it arises from a comprehensive rewiring of the entire immune regulatory network, which encompasses hyperactive innate immune responses and dysregulated formation of adaptive immune memory. Preclinical studies have demonstrated that probiotic strains such as *Pediococcus pentosaceus* CECT8330 exhibit remarkable immunomodulatory potential in juvenile murine colitis models (65). This strain suppresses NF-κB signaling activation in intestinal epithelial cells, thereby reducing reactive oxygen species (ROS) production and inhibiting epithelial apoptosis, ultimately restoring intestinal barrier function (65). More critically, it effectively “reprograms” macrophages from a pro-inflammatory M1 phenotype toward an anti-inflammatory M2 phenotype, while significantly reducing secretion of the pro-inflammatory cytokine IL-1β (65). These findings validate the feasibility of “immunological reprogramming” in PUC-namely, redirecting inflammatory immune responses toward immune homeostasis by modulating the local microenvironment, rather than simply blocking single inflammatory mediators.

With respect to the development of novel agents targeting immune pathways, research in pediatric populations remains in its exploratory phase (**Supplementary Table S1**). Monoclonal antibodies targeting the p19 subunit of interleukin-23 (IL-23)-such as risankizumab and mirikizumab-have demonstrated substantial efficacy in induction and maintenance therapy for moderate-to-severe UC in adults, promoting both clinical and endoscopic remission through blockade of IL-23-mediated Th17 immune responses (129, 130). However, the safety, pharmacokinetics, and long-term efficacy of these targeted therapies in children still require validation through dedicated pediatric clinical trials. Fecal microbiota transplantation (FMT) has shown nearly 100% cure rates in pediatric recurrent *Clostridioides difficile* infection and can effectively restore gut microbial diversity and function (131). Nevertheless, the efficacy of FMT in UC alone remains controversial, and large-scale pediatric randomized controlled trial evidence is lacking (127).

Moreover, recent advances in B-cell-directed immunotherapy have opened a new frontier in UC treatment. Historically, B cells have received less attention in UC therapeutics, partly due to the negative results of a phase II trial of rituximab, which failed to demonstrate clinical benefit in UC patients (132). However, a landmark case report published in the New England Journal of Medicine in 2025 demonstrated that CD19 CAR T-cell therapy achieved drug-free clinical remission and endoscopic mucosal healing in a young adult patient with severe, multidrug-resistant UC (133). The rationale for B-cell targeting was supported by the presence of autoantibodies against colonic αvβ6-integrin, which disrupt activation of the anti-inflammatory TGF-β pathway (134). Unlike monoclonal antibodies, CAR T cells exhibit superior tissue penetration and more profound depletion of tissue-resident B cells and plasma cells within mucosal niches (134). Although CAR T-cell therapy remains intensive and costly, with risks of cytokine release syndrome and neurotoxicity, its success provides proof of concept that B cells can act as key pathogenic drivers in a subset of UC patients (133, 134). In the pediatric context, while CAR T cells have not yet been evaluated in children with UC, their successful application in pediatric autoimmune diseases such as systemic lupus erythematosus suggests this approach may eventually be extended to carefully selected pediatric UC patients with refractory disease (135). Nevertheless, significant challenges remain, including the need to identify predictive biomarkers (such as autoantibodies against αvβ6-integrin) and establish appropriate dosing and timing in the developing immune system of children.

Collectively, the treatment of PUC is progressively transitioning from an adult-derived empirical approach toward individualized precision medicine grounded in age-specific immune mechanisms. Future research should focus on deepening basic investigations into the interplay between the intestinal immune microenvironment (e.g., macrophage polarization, Th17/Treg balance) and gut microbiota in pediatric pathogenesis, as well as conducting multicenter pediatric randomized controlled trials of novel targeted agents (e.g., IL-23 inhibitors, TL1A antibodies), integrating therapeutic drug monitoring and population pharmacokinetic modeling to optimize dosing regimens (130, 136).

Beyond these mechanistic studies, particular attention should be directed toward the prediagnostic phase of PUC. Recent advances have begun to characterize the molecular trajectories preceding IBD diagnosis, including alterations in serum metabolomic profiles, changes in gut microbial composition, and elevations in fecal calprotectin and other inflammatory markers detectable years before clinical onset (137–139). However, most of these prediagnostic studies have been conducted predominantly in adult populations, leaving a substantial gap in our understanding of whether and how the prediagnostic phase differs between pediatric-onset and adult-onset UC (140). Given that immune system maturation and gut microbiota assembly occur during critical developmental windows in childhood, the prediagnostic trajectory of PUC may be fundamentally distinct from that of adult-onset disease (140). Future longitudinal cohort studies tracking healthy children from birth through the development of UC, combined with multi-omics profiling of biospecimens collected at multiple time points before diagnosis, will be essential to elucidate the age-specific prediagnostic features of PUC and to identify early biomarkers that could enable timely intervention or even disease prevention (141, 142).

In summary, research on the pathogenesis of PUC is converging toward the elucidation of age-dependent immunological mechanisms. Future research endeavors are warranted to adopt integrated multi-omics analytical frameworks-encompassing genomics, single-cell transcriptomics, and microbiomics-placing specific focus on delineating the dynamic regulation of macrophage polarization, age-associated shifts in the Th17/Treg immune equilibrium, and the functional contributions of innate lymphoid cells (ILCs) to PUC progression. These systematic investigations will support the comprehensive elucidation of PUC pathogenic mechanisms and facilitate the identification of viable targeted strategies for immunotherapeutic intervention.
