# Supplementary material for: Immune–microbe interactions in pediatric ulcerative colitis: clinical features, pathogenesis, and novel targeted therapeutic strategies
Source: Front Immunol. 2026 Sep 14;17:1948185. doi: 10.3389/fimmu.2026.1948185 (PMC13617377; doi:10.3389/fimmu.2026.1948185)
Supplement: Supplementary file 1 [file Table1.docx]

**Table S1**. **Clinical trials of therapeutic agents for pediatric ulcerative colitis.**

| **NCT Number** | **Interventions** | **Conditions** | **Study Title** | **Brief Summary** | **Phases** | **Reference** |
| --- | --- | --- | --- | --- | --- | --- |
| NCT01585155 | DRUG: TA-650 | PUC | Clinical Study of TA-650 in Pediatric Patients With UC | The purpose of this study is to evaluate the the efficacy of TA-650 using Clinical activity index (CAI) score and other evaluation indicators in pediatric patients with moderate to severe UC after TA-650 administration of at a dose of 5 mg/kg at weeks 0, 2, and 6, and then every 8 weeks at weeks 14 and 22. The safety and pharmacokinetics are also evaluated. | PHASE3 | (1) |
| NCT05679622 | OTHER: Fecal Microbiota Transplantation (FMT) | UC | FMT in PUC | This study included two topics: one was to test the efficacy and safety of FMTs plus partial enteral nutrition (PEN) in refractory pediatric UC where conventional therapy has failed, and the other was to explore the efficacy and safety of FMT plus PEN as first-line therapy for pediatric active UC | NA | (2) |
| NCT06614387 | DRUG: Infliximab | UC | Vedolizumab in PUC- Comparison With Infliximab | This is a multicenter, double-blind, randomized study to compare the safety, effectiveness and immunogenicity (antibodies against drug) of two biological drugs administered intravenously - vedolizumab (a drug not registered in pediatric patients) and infliximab (used as standard therapy), in pediatric patients with UC aged 6-18 years. | PHASE3 | (3) |
| NCT05218850 | DRUG: Butyrate | UC | The Use of Butyrate Therapy in PUC | Butyrate is an important metabolite produced by the gut microbiome and has been shown as a helpful therapy in UC. This is a feasibility study to determine the efficacy of butryate enemas in PUC. | PHASE2 | (4) |
| NCT03582969 | OTHER: Fecal transplantation\|OTHER: Placebo | UC | Capsulized FMT in PUC Patients | The primary objective is to assess the safety and feasibility of multiple, oral, FMT, in newly diagnosed pediatric patients with mild-moderate UC. | PHASE2 | (5) |
| NCT00781638 | DEVICE: Adacolumn | UC | ADAPT Adacolumn Pediatric Trial in UC | Children aged up to 18 years with moderately active UC (PUCAI:35-64) will receive one weekly Adacolumn apheresis treatment over 5 consecutive weeks, followed by up to 3 optional Adacolumn apheresis treatments over 3 consecutive weeks. Primary end point is PUCAI at Week 12. | NA | (6) |
| NCT01536535 | DRUG: Mesalazine\|DRUG: IV Corticosteroid\|DRUG: Oral Corticosteroids\|OTHER: Additional Therapies\|PROCEDURE: Colectomy | UC | Predicting Response to Standardized Pediatric Colitis Therapy | This is a multi-center, open-label study to determine the safety and effectiveness (how well it works) of two standardized treatments called "mesalamine" (Pentasa) and "prednisone" in children with newly diagnosed UC. | PHASE4 | (7) |
| NCT02922881 | OTHER: UC Diet (UCD) | UC | Microbiota-targeted Diet for Pediatric UC | The primary objective of this study is to determine whether the UCD can induce remission or response in pediatric UC patients with active mild to moderate UC on a stable medication. | NA | (8) |
| NCT06260163 | DRUG: Guselkumab Subcutaneous\|DRUG: Guselkumab Intravenous | Colitis, Ulcerative | A Study of Guselkumab in Pediatric Participants With Moderately to Severely Active UC | The purpose of this study is to evaluate the efficacy of guselkumab in pediatric participants with moderately to severely active UC at the end of maintenance therapy among participants who were induction responders. | PHASE3 | (9) |
| NCT05076175 | DRUG: Ozanimod | Colitis, Ulcerative | A Study Investigating Oral Ozanimod (RPC1063) in Pediatric Participants With Moderate to Severe Active UC | The purpose of this study is to evaluate the effectiveness and safety of ozanimod (RPC1063) in achieving and maintaining clinical remission. Ozanimod will be administered orally to pediatric participants with moderate to severe active UC who have had an inadequate response to conventional therapy. | PHASE2\|PHASE3 | (10) |
| NCT02065557 | BIOLOGICAL: Adalimumab\|BIOLOGICAL: Placebo | UC | Efficacy and Safety of Adalimumab in Pediatric Subjects With Moderate to Severe UC | The purpose of the study is to demonstrate the efficacy and safety, and to assess the pharmacokinetics of adalimumab administered subcutaneously (SC) in pediatric subjects with moderate to severe UC. | PHASE3 | (11) |
| NCT02632175 | BIOLOGICAL: Adalimumab | UC | Long-term Safety and Efficacy Study of Adalimumab in Pediatric Subjects With UC | This study assesses the long-term safety and efficacy of adalimumab in pediatric subjects with UC. | PHASE3 | (12) |
| NCT07470879 | DRUG: Etrasimod | Colitis, Ulcerative | A Study of How the Medicine Called "Etrasimod" Works in Children With the Gut Disease Called UC | The purpose of this study is to determine the safety, efficacy, and pharmacokinetics (PK) of etrasimod for the treatment of moderately to severely active UC in pediatrics participants. | PHASE2 | (13) |
| NCT04004611 | DRUG: Mirikizumab | UC | A Study of Mirikizumab (LY3074828) in Children and Teenagers With UC | This study was designed to evaluate how the body processes and removes mirikizumab. The study also evaluated safety and disease response in pediatric participants with UC taking mirikizumab. | PHASE2 | (14) |
| NCT04630028 | DRUG: Ustekinumab Dose Based on BSA and Body Weight\|DRUG: Matching Placebo | Colitis, Ulcerative | A Study of Ustekinumab in Pediatric Participants With Moderately to Severely Active UC | The purpose of this study is to evaluate: a) the efficacy of ustekinumab dosing in inducing clinical remission, b) safety profile of ustekinumab, and c) ustekinumab exposure in pediatric participants with moderately to severely active UC. | PHASE3 | (15) |
| NCT05784246 | DRUG: Mirikizumab IV\|DRUG: Mirikizumab SC | UC | A Study of Mirikizumab (LY3074828) in Pediatric Participants With Moderately to Severely or Active UC | The main purpose of this study is to investigate efficacy, pharmacokinetics and safety of the drug in pediatric participants with moderately to severely active UC. | PHASE3 | (16) |
| NCT01947101 | BIOLOGICAL: FMT | UC | FMT for Treatment of UC in Children | The purpose of this study is to (1) examine the microbiome of stool and colon (2) and to determine whether healthy-donated stool enemas can treat PUC. (3) The investigators will also study the effects of the treatment on colonic gene expression. | PHASE1 | (17) |
| NCT05202990 | DRUG: FMT by Stool capsules\|DRUG: FMT by Intra-rectal enemas | PUC in Remission | Oral FMT in PUC | The purpose of this study is to evaluate whether FMT by frozen stool capsules in pediatric UC patients in remission after corticosteroid treatment, can modify their dysbiotic gut microbiota by increasing the richness of their microbiota at 6 months. | PHASE2 | (18) |
| NCT02093663 | DRUG: MMX Mesalamine/Mesalazine (Low Dose)\|DRUG: MMX Mesalamine/Mesalazine (High Dose) | UC | Safety and Efficacy of MMX Mesalamine/Mesalazine in Pediatric Subjects With Mild to Moderate UC | To assess clinical response to MMX mesalamine/mesalazine between a low and high dose in children and adolescents aged 5-17 years with mild to moderate UC or who are in remission. | PHASE3 | (19) |
| NCT03596645 | DRUG: Golimumab\|DRUG: Infliximab | Colitis, Ulcerative | A Study to Assess the Efficacy and Safety of Golimumab in Pediatric Participants With Moderately to Severely Active UC | The purpose of this study is to evaluate efficacy of golimumab in inducing clinical remission as assessed by the Mayo score, in pediatric participants with moderately to severely active UC. In addition, the safety profile of golimumab, in pediatric participants with moderately to severely active UC will be assessed. | PHASE3 | (20) |
| NCT05782907 | DRUG: Upadacitinib | UC | Study to Assess Adverse Events, Change in Disease Activity, and How Oral Upadacitinib Moves Through the Body of Pediatric Participants With Moderately to Severely Active UC. | Upadacitinib (RINVOQ) is a drug approved in adults for moderate- to severely active UC and is being developed for moderate- to severely active UC in pediatric participants. | PHASE3 | (21) |
| NCT06865417 | DRUG: Filgotinib | UC | A Study Evaluating the Effects of Filgotinib in Children and Teenagers With UC | The aim of this study is to evaluate the efficacy, safety, tolerability, and PK of filgotinib as a treatment for UC in children and adolescents aged from 8 to less than 18 years. | PHASE3 | (22) |
| NCT00336492 | BIOLOGICAL: infliximab\|BIOLOGICAL: infliximab\|BIOLOGICAL: infliximab\|BIOLOGICAL: infliximab | UC | A Study of the Safety and Efficacy of Infliximab (REMICADE ) in Pediatric Patients With Moderately to SeverelyActive UC | The purpose of the study is to evaluate the effectiveness and safety of infliximab (Remicade) in children with moderately to severely active UC. | PHASE3 | (23) |
| NCT01900574 | DRUG: Golimumab | Colitis, Ulcerative | A Study to Assess the Safety and Pharmacokinetics of Subcutaneously Administered Golimumab, a Human Anti-TNF Antibody, in Pediatric Patients With Moderate to Severe Active UC | The purpose of this study is to evaluate the pharmacokinetics (what the body does to the study medication) and safety of subcutaneously (under the skin) administered golimumab in pediatric participants (aged 2 to 17 years) with moderately to severely active UC. | PHASE1 | (24) |
| NCT05976802 | DRUG: High dose budesonide rectal foam\|DRUG: Low dose budesonide rectal foam\|DRUG: Matching placebo rectal foam | UC | Dose Ranging Study to Evaluate the Safety, Efficacy, and Pharmacokinetics of Budesonide Rectal Foam in Pediatric Patients Aged 5 to 17 Years With Active, Mild to Moderate Distal UC | The primary objective of this study is to evaluate the efficacy (as measured by induction of remission) of two dose levels (low and high) per age group of budesonide rectal foam as compared to an equivalent volume of rectally administered placebo foam over the same dosing schedule, in pediatric subjects with active, mild to moderate distal UC. | PHASE4 | (25) |
| NCT07071519 | DRUG: Risankizumab\|DRUG: Risankizumab | UC | A Study to Learn More About How Risankizumab Works in Young Participants With UC | This study will assess how Risankizumab moves through the body as well as how safe and effective it is in treating pediatric participants with moderate to severely active UC. | PHASE3 | (26) |
| NCT04624230 | DRUG: tofacitinib | UC | Evaluation of Oral Tofacitinib in Children Aged 2 to 17 Years Old Suffering From Moderate to Severe UC | This study, A3921210 is designed to evaluate the efficacy, safety and PK of tofacitinib in pediatric participants with moderately to severely active UC. | PHASE3 | (27) |
| NCT04779307 | DRUG: Vedolizumab | Colitis, Ulcerative | A Study of Vedolizumab in Children and Teenagers With Moderate to Severe UC | In this study, children and teenagers with moderate to severe UC will be treated with vedolizumab.The main aim of the study is to check if participants achieve remission after treatment with vedolizumab. | PHASE3 | (28) |
| NCT04999228 | DRUG: Infliximab\|DRUG: Corticosteroids | UC\|Infliximab\|Children | Top Down Versus Step up in PUC | Newly diagnosed moderate to severe pediatric UC patients (6-18 years old) will be randomly divided into infliximab (IFX) treatment group (Top down group, TD) and corticosteroids (CS) treatment group (Step-up group, SU). Mucosal healing rate at week 12 will be compared between the two groups. The relapse rates and sustained durations of remission within one year will also be evaluated. | PHASE4 | (29) |
| NCT03138655 | DRUG: Vedolizumab | UC\|CD | Vedolizumab IV in Pediatric Participants With UC or Crohn's Disease (CD) | The purpose of this study is to evaluate vedolizumab PK, safety and tolerability in pediatric participants with moderately to severely active UC or CD. | PHASE2 | (30) |
| NCT04844606 | DRUG: Mirikizumab\|DRUG: Mirikizumab | UC\|UC Chronic\|IBDs\|CD | A Master Protocol (AMAZ): A Study of Mirikizumab (LY3074828) in Pediatric Participants With UC or CD (SHINE-ON) | The main purpose of this study is to evaluate the long-term efficacy of mirikizumab in pediatric participants with UC or CD. | PHASE3 | (31) |
| NCT01201122 | DRUG: Mesalamine | Mild to Moderate UC | Once Versus Twice Daily Mesalamine to Induce Remission in PUC | The purpose of this study is to evaluate effectiveness of once daily dosing of Pentasa compared with twice daily in children with mild to moderate active UC. | PHASE4 | (32) |
| NCT02487238 | BIOLOGICAL: Fecal Microbiota Enema\|BIOLOGICAL: Normal Saline Enema | IBD\|UC | Pediatric FMT for UC | The PediFETCh study is a pilot trial designed to assess the feasibility of FMTs for the therapy of PUC and pediatric inflammatory bowel disease-unclassified (IBD-U). Investigators will test the hypothesis that a protocol of twice-weekly retention enemas delivered over six weeks, using fecal transplant material from a healthy donor, will improve clinical and biological disease markers in patients with pediatric UC or IBD-U. | PHASE1 | (33) |
| NCT05442567 | DRUG: Vedolizumab IV\|OTHER: No Intervention | UC\|CD | A Study of Vedolizumab in Children With UC or CD | The purpose of this study is to collect the long-term safety of vedolizumab in children with UC or CD. | PHASE3 | (34) |
| NCT03196427 | DRUG: Vedolizumab | UC\|CD | Long-term Safety With Vedolizumab Intravenous (IV) in Pediatric Participants With UC or CD | The purpose of this study is to determine the safety profile of long-term vedolizumab IV treatment in pediatric participants with UC or CD. | PHASE2 | (35) |
| NCT06100289 | DRUG: Vedolizumab IV\|DRUG: Vedolizumab SC | UC\|CD | A Study of Vedolizumab in Children and Teenagers With UC or CD | The main aim of this study is to learn how the body of a child or teenager with moderately to severely active UC or CD processes vedolizumab (pharmacokinetics) given just under the skin SC. | PHASE3 | (36) |
| NCT02330653 | BIOLOGICAL: FMT\|BIOLOGICAL: Placebo | IBDs\|UC\|Crohn Disease | FMT in Pediatric Active UC and Pediatric Active Crohn's Colitis | The primary aims of this phase I/II, randomized, placebo controlled study are the assessment of safety and tolerability of universal donor FMT compared to placebo in pediatric and young adult subjects (ages 5 years through 30 years) with active UC or active CD who have failed, are intolerant to, or have refused traditional first-line maintenance therapy. | PHASE1\|PHASE2 | (37) |
| NCT06777706 | DIETARY_SUPPLEMENT: QD (Qing Dai) | UC\|IBDs | Open Label Study to Assess Safety & Efficacy of QD for Induction of Remission in Pediatric Patients with UC | To assess the efficacy and safety of QD as induction therapy in pediatric patients with mild-moderate active UC. | NA | (38) |
| NCT05092269 | DRUG: Ustekinumab | CD\|Colitis, Ulcerative\|Arthritis, Psoriatic | A Long-term Extension Study of Ustekinumab in Pediatric Participants | The purpose of this study is to collect long-term safety data of subcutaneous (SC) ustekinumab | PHASE3 | (39) |
| NCT00720538 | DRUG: Thalidomide\|DRUG: placebo | IBDs\|CD\|UC | Thalidomide in Pediatric IBDs. | This is a randomized placebo controlled (RCT) double blind study, to evaluate the efficacy of thalidomide in inducing clinical remission at 8 weeks in refractory IBD patients aged 2-20 years. | PHASE3 | (40) |
| NCT00889161 | DRUG: Curcumin | IBD\|UC\|CD | Curcumin in Pediatric IBD | This is a single center, open label forced dose titration study designed to determine the tolerability of curcumin in pediatric patients with IBD. | PHASE1 | (41) |
| NCT00606346 | BIOLOGICAL: Anti TNF therapy including infliximab\|DRUG: No Biologics | CD\|UC\|Indeterminate Colitis\|IBDs | A Multicenter, Prospective, Long-term, Observational Registry of Pediatric Patients With IBD | This is a single center, open label forced dose titration study designed to determine the tolerability of curcumin in pediatric patients with IBD. |  | (42) |
| NCT02862132 | DRUG: Vedolizumab | CD\|UC\|IBD | Predicting Response to Vedolizumab in Pediatric IBDs | The investigators aim to prospectively explore the real life short and longer term outcomes of VDZ in pediatric IBD (including growth) and to develop a prediction model for treatment success based on VDZ trough levels and other clinical and laboratory variables. | NA | (43) |
| NCT01757964 | BIOLOGICAL: Bacteriotherapy | IBD\|CD\|UC\|CD | Bacteriotherapy in Pediatric IBD | In this study, the investigators want to see if Bacteriotherapy (also referred to as stool transplantation) improves the symptoms and decreases inflammation in children with IBD. | PHASE1 | (44) |
| NCT03847467 | DRUG: 2'-Fucosyllactose\|OTHER: Placebo | IBDs\|CD\|UC | Pilot and Feasibility Study of 2'-FL as a Dietary Supplement in IBD Patients Receiving Stable Maintenance Anti-TNF Therapy | Randomized, placebo-controlled dose-ranging study of 2'-FL in IBD, CD and UC. | PHASE1\|PHASE2 | (45) |
| NCT02636517 | BIOLOGICAL: FMT | Clostridium Difficile\|IBD\|CD\|UC | Fecal Microbiome Transplant | The aims of this study are to determine the safety and efficacy of FMT treatment in pediatric patients with recurrent or moderate to severe C. Difficile without (through an observational study) and with (through a clinical trial) IBD and to determine the effect of FMT on the gut microbiota through the use of 454 pyrosequencing before and after transplantation in these patients. | PHASE1 | (46) |
| NCT05456763 | DIETARY_SUPPLEMENT: sodium butyrate\|OTHER: placebo | IBDs | Butyrate in Pediatric IBD | The first prospective randomised placebo-controlled study in the IBD pediatric population was conducted to evaluate the effectiveness of oral sodium butyrate as add-on therapy in IBD. | NA | (47) |
| NCT01560819 | DRUG: Gut Microbial Transplantation | IBD | Gut Microbial Transplantation in Pediatric IBDs | This research will be conducted in the Helen DeVos Children's Hospital (HDVCH) Pediatric gastrointestinal outpatient clinic. A pilot study of ten patients will be conducted to evaluate if GMT improves clinical symptoms in patients with IBD. | PHASE1 | (48) |
| NCT02150551 | BIOLOGICAL: Allogeneic bone marrow-derived mesenchymal stromal cells | IBDs | Safety and Tolerability Of Allogeneic Mesenchymal Stromal Cells in Pediatric IBD | In this trial, investigators will infuse donor bone marrow mesenchymal stromal cells intravenously, as a treatment for pediatric CD or UC that has not responded to conventional therapies. The goals of this study are to test the safety and tolerability of donor mesenchymal stromal cells in children with IBD. | PHASE1 | (49) |

References：

1.	NCT01585155. Clinical Study of Ta-650 in Pediatric Patients with Ulcerative Colitis. Available from: <https://clinicaltrials.gov/study/NCT01585155>.

2.	NCT05679622. Fecal Microbiota Transplantation in Pediatric Ulcerative Colitis (Uc). Available from: <https://clinicaltrials.gov/study/NCT05679622>.

3.	NCT06614387. Vedolizumab in Pediatric Ulcerative Colitis- Comparison with Infliximab. Available from: <https://clinicaltrials.gov/study/NCT06614387>.

4.	NCT05218850. The Use of Butyrate Therapy in Pediatric Ulcerative Colitis. Available from: <https://clinicaltrials.gov/study/NCT05218850>.

5.	NCT03582969. Capsulized Fecal Microbiota Transplantation in Pediatric Ulcerative Colitis Patients. Available from: <https://clinicaltrials.gov/study/NCT03582969>.

6.	NCT00781638. Adapt Adacolumn Pediatric Trial in Ulcerative Colitis. Available from: <https://clinicaltrials.gov/study/NCT00781638>.

7.	NCT01536535. Predicting Response to Standardized Pediatric Colitis Therapy. Available from: <https://clinicaltrials.gov/study/NCT01536535>.

8.	NCT02922881. Microbiota-Targeted Diet for Pediatric Uc. Available from: <https://clinicaltrials.gov/study/NCT02922881>.

9.	NCT06260163. A Study of Guselkumab in Pediatric Participants with Moderately to Severely Active Ulcerative Colitis. Available from: <https://clinicaltrials.gov/study/NCT06260163>.

10.	NCT05076175. A Study Investigating Oral Ozanimod (Rpc1063) in Pediatric Participants with Moderate to Severe Active Ulcerative Colitis. Available from: <https://clinicaltrials.gov/study/NCT05076175>.

11.	NCT02065557. Efficacy and Safety of Adalimumab in Pediatric Subjects with Moderate to Severe Ulcerative Colitis. Available from: <https://clinicaltrials.gov/study/NCT02065557>.

12.	NCT02632175. Long-Term Safety and Efficacy Study of Adalimumab in Pediatric Subjects with Ulcerative Colitis. Available from: <https://clinicaltrials.gov/study/NCT02632175>.

13.	NCT07470879. A Study of How the Medicine Called "Etrasimod" Works in Children with the Gut Disease Called Ulcerative Colitis. Available from: <https://clinicaltrials.gov/study/NCT07470879>.

14.	NCT04004611. A Study of Mirikizumab (Ly3074828) in Children and Teenagers with Ulcerative Colitis (Uc). Available from: <https://clinicaltrials.gov/study/NCT04004611>.

15.	NCT04630028. A Study of Ustekinumab in Pediatric Participants with Moderately to Severely Active Ulcerative Colitis (Uc). Available from: <https://clinicaltrials.gov/study/NCT04630028>.

16.	NCT05784246. A Study of Mirikizumab (Ly3074828) in Pediatric Participants with Moderately to Severely or Active Ulcerative Colitis. Available from: <https://clinicaltrials.gov/study/NCT05784246>.

17.	NCT01947101. Fecal Microbiota Transplantation (Fmt) for Treatment of Ulcerative Colitis in Children. Available from: <https://clinicaltrials.gov/study/NCT01947101>.

18.	NCT05202990. Oral Fecal Microbiota Transplantation in Pediatric Ulcerative Colitis. Available from: <https://clinicaltrials.gov/study/NCT05202990>.

19.	NCT02093663. Safety and Efficacy of Mmx Mesalamine/Mesalazine in Pediatric Subjects with Mild to Moderate Ulcerative Colitis. Available from: <https://clinicaltrials.gov/study/NCT02093663>.

20.	NCT03596645. A Study to Assess the Efficacy and Safety of Golimumab in Pediatric Participants with Moderately to Severely Active Ulcerative Colitis. Available from: <https://clinicaltrials.gov/study/NCT03596645>.

21.	NCT05782907. Study to Assess Adverse Events, Change in Disease Activity, and How Oral Upadacitinib Moves through the Body of Pediatric Participants with Moderately to Severely Active Ulcerative Colitis. Available from: <https://clinicaltrials.gov/study/NCT05782907>.

22.	NCT06865417. A Study Evaluating the Effects of Filgotinib in Children and Teenagers with Ulcerative Colitis. Available from: <https://clinicaltrials.gov/study/NCT06865417>.

23.	NCT00336492. A Study of the Safety and Efficacy of Infliximab(Remicade ) in Pediatric Patients with Moderately to Severelyactive Ulcerative Colitis. Available from: <https://clinicaltrials.gov/study/NCT00336492>.

24.	NCT01900574. A Study to Assess the Safety and Pharmacokinetics of Subcutaneously Administered Golimumab, a Human Anti-Tnf伪 Antibody, in Pediatric Patients with Moderate to Severe Active Ulcerative Colitis. Available from: <https://clinicaltrials.gov/study/NCT01900574>.

25.	NCT05976802. Dose Ranging Study to Evaluate the Safety, Efficacy, and Pharmacokinetics of Budesonide Rectal Foam in Pediatric Patients Aged 5 to 17 Years with Active, Mild to Moderate Distal Ulcerative Colitis. Available from: <https://clinicaltrials.gov/study/NCT05976802>.

26.	NCT07071519. A Study to Learn More About How Risankizumab Works in Young Participants with Ulcerative Colitis. Available from: <https://clinicaltrials.gov/study/NCT07071519>.

27.	NCT04624230. Evaluation of Oral Tofacitinib in Children Aged 2 to 17 Years Old Suffering from Moderate to Severe Ulcerative Colitis. Available from: <https://clinicaltrials.gov/study/NCT04624230>.

28.	NCT04779307. A Study of Vedolizumab in Children and Teenagers with Moderate to Severe Ulcerative Colitis (Uc). Available from: <https://clinicaltrials.gov/study/NCT04779307>.

29.	NCT04999228. Top down Versus Step up in Pediatric Ulcerative Colitis. Available from: <https://clinicaltrials.gov/study/NCT04999228>.

30.	NCT03138655. Vedolizumab Iv in Pediatric Participants with Ulcerative Colitis (Uc) or Crohn's Disease (Cd). Available from: <https://clinicaltrials.gov/study/NCT03138655>.

31.	NCT04844606. A Master Protocol (Amaz): A Study of Mirikizumab (Ly3074828) in Pediatric Participants with Ulcerative Colitis or Crohn's Disease (Shine-on). Available from: <https://clinicaltrials.gov/study/NCT04844606>.

32.	NCT01201122. Once Versus Twice Daily Mesalamine to Induce Remission in Pediatric Ulcerative Colitis. Available from: <https://clinicaltrials.gov/study/NCT01201122>.

33.	NCT02487238. Pediatric Fecal Microbiota Transplant for Ulcerative Colitis. Available from: <https://clinicaltrials.gov/study/NCT02487238>.

34.	NCT05442567. A Study of Vedolizumab in Children with Ulcerative Colitis (Uc) or Crohn's Disease (Cd). Available from: <https://clinicaltrials.gov/study/NCT05442567>.

35.	NCT03196427. Long-Term Safety with Vedolizumab Intravenous (Iv) in Pediatric Participants with Ulcerative Colitis (Uc) or Crohn's Disease (Cd). Available from: <https://clinicaltrials.gov/study/NCT03196427>.

36.	NCT06100289. A Study of Vedolizumab in Children and Teenagers with Ulcerative Colitis or Crohn's Disease. Available from: <https://clinicaltrials.gov/study/NCT06100289>.

37.	NCT02330653. Fecal Microbiota Transplant (Fmt) in Pediatric Active Ulcerative Colitis and Pediatric Active Crohn's Colitis. Available from: <https://clinicaltrials.gov/study/NCT02330653>.

38.	NCT06777706. Open Label Study to Assess Safety & Efficacy of Qd for Induction of Remission in Pediatric Patients with Uc. Available from: <https://clinicaltrials.gov/study/NCT06777706>.

39.	NCT05092269. A Long-Term Extension Study of Ustekinumab in Pediatric Participants. Available from: <https://clinicaltrials.gov/study/NCT05092269>.

40.	NCT00720538. Thalidomide in Pediatric Inflammatory Bowel Diseases. Available from: <https://clinicaltrials.gov/study/NCT00720538>.

41.	NCT00889161. Curcumin in Pediatric Inflammatory Bowel Disease. Available from: <https://clinicaltrials.gov/study/NCT00889161>.

42.	NCT00606346. A Multicenter, Prospective, Long-Term, Observational Registry of Pediatric Patients with Inflammatory Bowel Disease. Available from: <https://clinicaltrials.gov/study/NCT00606346>.

43.	NCT02862132. Predicting Response to Vedolizumab in Pediatric Inflammatory Bowel Diseases. Available from: <https://clinicaltrials.gov/study/NCT02862132>.

44.	NCT01757964. Bacteriotherapy in Pediatric Inflammatory Bowel Disease. Available from: <https://clinicaltrials.gov/study/NCT01757964>.

45.	NCT03847467. Pilot and Feasibility Study of 2'-Fl as a Dietary Supplement in Ibd Patients Receiving Stable Maintenance Anti-Tnf Therapy. Available from: <https://clinicaltrials.gov/study/NCT03847467>.

46.	NCT02636517. Fecal Microbiome Transplant. Available from: <https://clinicaltrials.gov/study/NCT02636517>.

47.	NCT05456763. Butyrate in Pediatric Inflammatory Bowel Disease. Available from: <https://clinicaltrials.gov/study/NCT05456763>.

48.	NCT01560819. Gut Microbial Transplantation in Pediatric Inflammatory Bowel Diseases. Available from: <https://clinicaltrials.gov/study/NCT01560819>.

49.	NCT02150551. Safety and Tolerability of Allogeneic Mesenchymal Stromal Cells in Pediatric Inflammatory Bowel Disease. Available from: <https://clinicaltrials.gov/study/NCT02150551>.
